# Role of Protein Arginine Methyltransferases and Inflammation in Muscle Pathophysiology

**DOI:** 10.3389/fphys.2021.712389

**Published:** 2021-08-19

**Authors:** Hyun-Kyung So, Sunghee Kim, Jong-Sun Kang, Sang-Jin Lee

**Affiliations:** ^1^Molecular Cell Biology, Single Cell Network Research Center, Sungkyunkwan University School of Medicine, Suwon, South Korea; ^2^Research Institute of Aging-Related Disease, AniMusCure Inc., Suwon, South Korea

**Keywords:** muscle, PRMTs, inflammation, cytokine, inflammation-related muscle disease, myokine, IL-6, exercise

## Abstract

Arginine methylation mediated by protein arginine methyltransferases (PRMTs) is a post-translational modification of both histone and non-histone substrates related to diverse biological processes. PRMTs appear to be critical regulators in skeletal muscle physiology, including regeneration, metabolic homeostasis, and plasticity. Chronic inflammation is commonly associated with the decline of skeletal muscle mass and strength related to aging or chronic diseases, defined as sarcopenia. In turn, declined skeletal muscle mass and strength can exacerbate chronic inflammation. Thus, understanding the molecular regulatory pathway underlying the crosstalk between skeletal muscle function and inflammation might be essential for the intervention of muscle pathophysiology. In this review, we will address the current knowledge on the role of PRMTs in skeletal muscle physiology and pathophysiology with a specific emphasis on its relationship with inflammation.

## Introduction

Aging is generally associated with numerous changes that may affect health and life span. One of the major problems among the elderly population is a progressive decline in skeletal muscle mass, strength, and functionality, defined as sarcopenia. This multifactorial pathological condition contributes to a reduction in functional capacity and an increase in the risk for developing secondary chronic diseases, such as metabolic diseases, chronic inflammation, and cardiovascular diseases (Dutta, 1997; Wang et al., 2014; Brook et al., 2016). Elderly people suffering from sarcopenia have an increased risk of adverse outcomes such as physical disability, injuries, frailty, social exclusion, and hospitalization, associated with increased mortality rate (Dutta, 1997; Visser and Schaap, 2011). Skeletal muscle (referred to as muscle hereafter) is the largest organ in humans, which constitutes about 30–40% of the body weight and plays critical roles in locomotion, energy expenditure, and glucose disposal (Reid and Fielding, 2012; Brook et al., 2016). Muscles of healthy adults display resilient adaptation capacity in energy metabolism and contractile function in response to diverse demands such as exercise, hormones, and nutritional states. Impairments in muscle metabolism and function have been implicated in metabolic pathologies such as glucose intolerance, insulin resistance, and obesity (Zurlo et al., 1990).

The progressive loss of muscle mass and strength is a hallmark of muscle aging, leading to reduced functional capacity and an increased risk of developing chronic metabolic diseases (Goodpaster et al., 2006; Sakuma and Yamaguchi, 2012). Muscle atrophy is triggered by an imbalance between protein synthesis and protein degradation by the ubiquitin–proteasome system (UPS), causing a decrease in muscle fiber size (Lecker et al., 2006). Diverse signaling pathways have been implicated in the control of muscle maintenance. Myostatin, also known as growth differentiation factor 8, is a member of the transforming growth factor-β family and a negative regulator of muscle growth. Myostatin–Smad2/3 axis inhibits Akt-dependent protein synthesis and growth of mature muscle cells (Taylor et al., 2001; Elkina et al., 2011). Activated Smad2/3 and p38MAPK are implicated in protein degradation in muscle cells *via* induction of Muscle RING-finger containing protein-1 (MuRF1) and Atrogin-1, two major muscle-specific E3 ubiquitin ligases (Glass, 2010) that are bonafide markers of muscle atrophy phenotype (Bodine et al., 2001). In addition to the imbalance in protein metabolism, decreased regenerative capacities of muscle stem cells have been also associated with muscle atrophy related to aging-related muscle diseases (Vandromme et al., 2001; Lecker et al., 2006; Bowen et al., 2015). Akt is a well-known promyogenic signaling in myogenic differentiation (Fujio et al., 2001) and plays a critical role in myoblast proliferation and survival induced by insulin-like growth factors (Lawlor and Rotwein, 2000). Akt/mTOR (mammalian target of rapamycin) signaling plays an essential role in the induction of protein synthesis and muscle hypertrophy. In addition, Akt/mTOR signaling inhibits the transcriptional function of Forkhead box O (FoxO), which induces MuRF1 and Atrogin-1 associated with protein degradation (Brunet et al., 1999; Saxton and Sabatini, 2017).

In addition to aging, local or systemic inflammation is closely associated with muscle wasting that is characteristic of several pathological states and is attributed to perturbed muscle protein metabolism (Muscaritoli et al., 2010; Costamagna et al., 2015; Sharma and Dabur, 2020). In this regard, results obtained from both the experimental models and human pathology have demonstrated that systemic inflammation is associated with reduced rates of protein synthesis concurrent with enhanced protein breakdown, accounting for muscle wasting (Espat et al., 1995). Chronic inflammation affects the muscle in diverse muscle diseases including muscular dystrophy and myopathies, cancer-induced muscle atrophy, and sarcopenia. In patients with cachectic cancer, several proinflammatory cytokines are elevated, and inflammation is considered as one of the diagnostic hallmarks of cachexia with extensive and progressive muscle wasting (Seruga et al., 2008; Fearon et al., 2011). In various models, cytokines such as tumor necrosis factor-alpha (TNFα), interleukin 6 (IL-6), IL-1, and interferon gamma (INF-γ) have been shown to be pro-cachectic inflammatory mediators (Espat et al., 1995; Cai et al., 2004). Among signaling events, NF-κB, which is the key transcription factor in cytokine-mediated signaling, plays a nodal role in muscle wasting. A study on the muscle-specific transgenic overexpression of activated IKKβ in mice reveals that the activation of NF-κB causes profound muscle wasting associated with elevated MuRF1, mimicking the clinical findings in cancer cachexia (Cai et al., 2004). So far, it has been concluded that muscle wasting caused by inflammation is mainly due to an increase in catabolic muscle protein breakdown through the activation of NF-κB and regulation of MuRF1 (Tisdale, 2002; Cai et al., 2004). However, the precise mechanism by which inflammation modulates the protein turnover rates is still not fully understood.

One critical event that causes muscle wasting is the denervation of muscle fibers associated with motor neuron degeneration or spinal cord injuries. Various cellular stress pathways, including chronic inflammation and mitochondrial dysfunction, have been implicated in motor neuron degeneration and muscle atrophy. Neuromuscular activity triggered by physical exercise induces muscle remodeling and production of factors such as cytokines, peptides, growth factors, and small organic molecules, called myokines (Schnyder and Handschin, 2015). Myokines can function in an auto-, para-, and endocrine fashion to modulate the target organs (Pedersen and Akerstrom, 2007). Accumulating evidence underlines the importance of myokines in the control of muscle and other organ functions (Chen et al., 2021). About 600 myokines have been identified to date, including myostatin, various interleukins (IL-6, IL-7, IL-8, and IL-15), irisin, fibroblast growth factor 21, brain-derived neurotrophic factor, insulin-like growth factor-1 (IGF-1), and leukemia inhibitory factor (Lee and Jun, 2019). Myokines appear to exert negative and positive effects on target organ function and metabolism, including the muscles, adipose tissue, bones, liver, pancreas, kidneys, and the brain. The decline in muscle function and plasticity perturbs myokine production, thereby contributing to changes in inflammatory responses and reduced body functionality. Inflammation is a nodal modulator of muscle plasticity and function under physiological conditions. Upon injury, inflammatory cytokines play critical roles in the removal of damaged myofibers and satellite cell function to ensure efficient muscle regeneration. However, in diverse disease conditions, chronic inflammation is associated with the deregulation of catabolic pathways in the muscles, leading to muscle wasting. Thus, understanding the molecular mechanisms that control muscle function and remodeling is pivotal for the development of therapeutic strategies to treat muscle and metabolic diseases associated with chronic inflammation.

In this review, we summarize the current knowledge on the role of protein arginine methyltransferases (PRMTs) in muscle physiology and pathophysiology with the specific emphasis on its relationship with inflammation.

## Protein Arginine Methyltransferases

**FIGURE 1:**
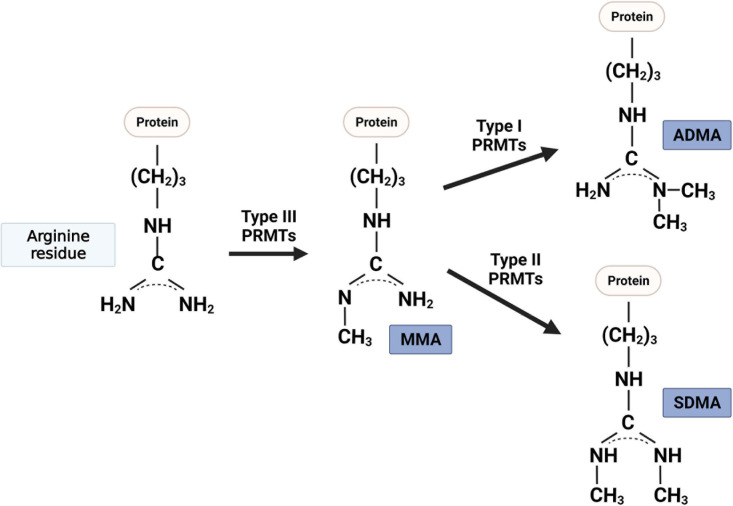
Protein arginine methyltransferases (PRMT) that methylate the nitrogen of specific arginine residues in proteins are classified into three types: (i) type I PRMTs (i.e., PRMT1, PRMT2, PRMT3, PRMT4, PRMT6, and PRMT8) catalyze the asymmetric NG, NG-dimethylarginine (ADMA); (ii) type II PRMTs (i.e., PRMT5 and PRMT9) catalyze the symmetric NG, NNG-dimethylarginine (ADMA); and (iii) type III PRMT (i.e., PRMT7) generates one methyl group to one side of nitrogen of arginine residue (MMA).

Post-translational modifications such as phosphorylation, ubiquitination, and lysine methylation of proteins play critical roles in a variety of biological processes, including muscle regeneration and metabolic homeostasis (Santos and Lindner, 2017). Arginine methylation is a newly emerging post-translational modification of histone or non-histone proteins, which modulates gene expression or signaling pathways in diverse biological processes. PRMTs catalyze the transfer of a methyl group from S-adenosyl-L-methionine to the guanidino nitrogen atoms of arginine on target proteins, thereby altering the stability, localization, and/or activity of their substrates (Paik and Kim, 1968; Bedford and Richard, 2005). There are three main forms of methylarginines identified in eukaryotes (Figure 1): mono-methylarginine (MMA), asymmetric dimethylarginine (ADMA), and symmetric dimethylarginine (SDMA) (Bedford, 2007). PRMT family consists of three categories based on their catalytic activity (Bedford and Clarke, 2009). Type I PRMTs [protein arginine methyltransferase 1 (PRMT1), PRMT2, PRMT3, PRMT4 (also called co-activator-associated arginine methyltransferase 1 (CARM1), PRMT6, and PRMT8) and type II PRMTs (PRMT5 and PRMT9)] mediate the formation of MMA as an intermediate before the establishment of ADMA or SDMA, respectively (Blanc and Richard, 2017; Stouth et al., 2017). PRMT7 is a type III enzyme that catalyzes only the formation of MMA (Feng et al., 2013). As mentioned above, arginine methylation is one of the histone modification marks modulating gene expression. In addition, PRMTs can also modify multiple non-histone substrates involved in biological events including transcription/translation, pre-mRNA splicing, cell signaling, DNA damage, receptor trafficking, and protein stability control (Blanc and Richard, 2017).

### Roles of PRMTs in Muscle Regeneration and Metabolism

Muscle regeneration is a multi-step process, involving activation of quiescent satellite cells, expansion of activated muscle progenitors, and terminal differentiation, followed by fusion of myoblasts into pre-existing myofibers (Brack and Rando, 2012). The maintenance of regenerative capacity appears to be critical for muscle homeostasis, and impaired satellite cell function is associated with muscle pathophysiology. In addition, muscle exhibits a remarkable adaptation capacity in energy metabolism and contractile functions in response to various stimuli such as exercise, hormones, and nutritional states (Zurlo et al., 1990). Muscle metabolic characteristics are modulated dynamically through glucose transport, mitochondrial biogenesis, and protein turnover (Bonaldo and Sandri, 2013).

Recent studies have demonstrated PRMTs as critical regulators of satellite cell function and muscle plasticity (Stouth et al., 2017). *In vitro* and *in vivo* studies have shown that PRMTs can stimulate or suppress factors important for muscle differentiation and muscle metabolic remodeling. The majority of current research on the role of PRMT in muscle is focused on PRMT1, PRMT4, PRMT5, and PRMT7, and only a few recent studies have demonstrated the *in vivo* function of these PRMTs by using gene-ablation mouse models.

#### Protein Arginine Methyltransferase 1

Protein arginine methyltransferase 1 is a predominant PRMT that accounts for ∼85% of cellular arginine methylation (Bedford and Clarke, 2009). The ablation of PRMT1 in mice results in early embryonic lethality around embryonic day 6.5, suggestive of its vital role for embryonic development (Yu et al., 2009). Recent studies reveal the vital role of ablation of PMRT1 in muscle stem cell function and plasticity (Stouth et al., 2018). The ablation of PRMT1 in satellite cells attenuates MyoD induction in muscle regeneration resulting in perturbed muscle regeneration (Blanc et al., 2017). PRMT1 appears to regulate MyoD levels through methylation of Six1, a co-activator of MyoD, and Eya1/Six1 recruitment at the MyoD promoter. Consequently, PRMT1-null satellite cells exhibit impaired differentiation accompanied by an increase in proliferating Pax7-positive cells (Blanc et al., 2017). On the other hand, PRMT1 seems to play critical roles in muscle homeostasis by modulation of FoxO3 and autophagy (Choi et al., 2019). PRMT1 ablation in muscles results in premature muscle atrophy caused by dysregulation of the PRMT6-FOXO3 axis, which contributes to elevated autophagy and upregulation of MuRF1 and Atrogin. These results signify the importance of PRMT1 in the control of muscle maintenance. Similar to these reports, the expression of PRMT1 and PRMT4 is altered in denervation-induced atrophy or muscular dystrophic muscles, suggesting that these PRMTs are involved in the atrophy process (Stouth et al., 2018). Furthermore, PRMT1-mediated methylation serves as a positive modulator of insulin receptor/insulin receptor substrate-1 (IRS-1)/ phosphatidylinositol-3 kinase (PI3K) pathway and the subsequent glucose uptake in muscle cells (Iwasaki and Yada, 2007).

Recent studies have proposed potential roles of PRMT1 in motor neuron pathophysiology related to muscle wasting. One of the major genes implicated in familial amyotrophic lateral sclerosis (ALS) is fused in sarcoma (FUS), a DNA/RNA binding protein, and multiple mutations in FUS have been identified (Rabbitts et al., 1993). FUS mutation causes enhanced formation/accumulation of FUS-RNA-PRMT1 complexes affecting multiple cellular events including abnormal neurite morphology resembling the defect observed in the brain tissue of patients with ALS (Jun et al., 2017). The mutation of FUS-R521C elicits the cytosolic accumulation of FUS/PRMT1 complex enhancing neurite degeneration. The arginine methylation of FUS by PRMT1 appears to regulate its cellular localization and the resulting stress granule formation. Cellular toxicities caused by ALS-linked FUS mutants underline the essential role of PRMT1-mediated arginine methylation of FUS for its physiological function (Tradewell et al., 2012; Yamaguchi and Kitajo, 2012). A recent report has demonstrated that FUS regulates acetylcholine receptor expression in subsynaptic myonuclei, and FUS mutation can cause muscle-intrinsic toxicity, likely contributing to ALS-related motor neuron degeneration (Picchiarelli et al., 2019). Thus, it is conceivable that PRMT1 and FUS might be involved in neuromuscular interaction that is critical for muscle homeostasis. All these results together suggest the prominent roles of PRMT1 in the maintenance of muscle mass and function. Further studies will be required to elucidate the distinct role of PRMT1 in muscle homeostasis and neuromuscular activity.

#### Co-activator-Associated Arginine Methyltransferase 1/Protein Arginine Methyltransferase 4

In addition to PRMT1, PRMT4 is the major ADMA-mediating enzyme in muscle (Stouth et al., 2017). PRMT4 can methylate Pax7 which acts as a molecular switch controlling the induction of Myf5 during the asymmetric division of satellite cells and the subsequent entry into the myogenic program (Kawabe et al., 2012). PRMT4 appears to be required for the later stages of myogenesis for the binding of SWI/SNF Brg1 ATPase chromatin remodeling enzymes and myogenin to the myogenin promoter (Dacwag et al., 2009). In addition, PRMT4 facilitates chromatin-remodeling mediated by the SWI/SNF complex to induce the expression of genes involved in glycogen metabolism in muscle cells (Wang D.S. et al., 2012). In contrast to the beneficial role of PRMT4 in muscle differentiation and regeneration, PRMT4 has also been implicated in muscle wasting (Liu et al., 2019). PRMT4 induces autophagy-related protein degradation by mediating FoxO3 activity. In support of this notion, recent studies in non-muscle cells have shown that PRMT4-dependent histone arginine methylation is an essential nuclear regulation to induce autophagy after nutrient starvation (Shin et al., 2016; Yu et al., 2020). Although the phenotype studies suggest an opposing role of PRMT1 and PRMT4 in the control of autophagy, the functional interaction between PRMT1 and PRMT4 in muscle homeostasis and plasticity is currently unclear. Both proteins seem to exert their effect through FoxO3 to increase or decrease autophagy, implying a crosstalk between these PRMTs. Further studies are needed to clarify this crosstalk in the modulation of muscle protein metabolism.

#### Protein Arginine Methyltransferase 5

The *in vitro* studies on PRMT5 in muscle cells demonstrate its importance in chromatin remodeling and the induction of myogenin associated with myoblast differentiation. PRMT5 is required for BRG1/MyoD-dependent chromatin remodeling and myogenic gene activation for differentiation (Dacwag et al., 2007). Similar to PRMT4, PRMT5 regulates myogenic gene activities through association with the BRG1 ATPase subunit of SWI/SNF chromatin remodeling enzymes (Dacwag et al., 2009). While PRMT5 is required for the early MyoD expression, it seems to be dispensable for the subsequent expression of myogenin and MEF2D. In addition, a PRMT5-interacting protein, called the cooperator of PRMT5 (COPR5), plays a role in myoblast differentiation through the regulation of cell cycle regulators (Paul et al., 2012). COPR5-depleted C2C12 cells exhibit very low levels of myosin heavy chain 1 and a failure to form multinucleated myotubes. The ablation of PRMT5 in satellite cells abrogates muscle regeneration associated with attenuated stem cell expansion. PRMT5 deficiency causes declined symmetric dimethylation of histone H3 at arginine 8 (H3R8me2s) in the p21 promoter region, leading strong upregulation of p21 expression which in turn negatively affects proliferation and differentiation of activated adult muscle stem cells (Zhang et al., 2015). These reports support the important role of PRMT5 in muscle stem cell function and regeneration. Further studies are required to elucidate the role of PRMT5 in muscle metabolism and homeostasis.

#### Protein Arginine Methyltransferase 7

Recent studies have proposed important roles of PRMT7 in muscle regenerative capacity and metabolism (Kim et al., 2019). PRMT7 ablation in satellite cells causes defective muscle regeneration due to premature cellular senescence of satellite cells (Blanc et al., 2016). PRMT7 deficiency in satellite cells decreases the expression of DNA methyltransferase 3b (DNMT3b) contributing to the hypomethylation of p21 promoter and the consequent increase of p21 expression. Thus, these data imply that PRMT7 is required to regulate the DNMT3b/p21 axis to control muscle stem cell regenerative capacity. In addition, we have recently shown that PRMT7 stimulates MyoD-mediated myoblast differentiation through p38MAPK methylation at arginine residue 70 (Jeong et al., 2020). The arginine-to-alanine mutation in p38MAPK alpha attenuates the heterodimerization of MyoD/E47 and the recruitment of Prmt7, MyoD, and Baf60c to the myogenin promoter leading to decreased myogenin expression. Furthermore, PRMT7 has also been implicated in muscle oxidative metabolism through activation of p38MAPK/ATF2/PGC1α pathways, contributing to enhanced mitochondrial biogenesis and function (Jeong et al., 2016). PRMT7 deficient mice exhibit reduced energy expenditure and induced age-dependent obesity. The metabolic regulation between lipid and glycolytic oxidation plays a critical role for the control of satellite cell states during regeneration (Nalbandian et al., 2020). Thus, PRMT7 could also be involved in satellite cell metabolic shift to ensure efficient myogenic activation and this needs to be investigated.

## Inflammation in Muscle Homeostasis

In physiological conditions, inflammation is a pivotal contributor for muscle regeneration and muscle homeostasis (Costamagna et al., 2015). In normal conditions, inflammatory cytokines are required to maintain the balance between anabolic and catabolic pathways to maintain muscle integrity and function. The modulation in acute events, such as muscle injury-triggered regeneration or a transient atrophic condition, is different from that in chronic events, such as long-term inflammatory processes affecting muscles occurring in genetic diseases, cancer-induced muscle atrophy, or sarcopenia. In these pathological conditions, proinflammatory cytokines induce catabolic pathways that impair muscle homeostasis and function, eventually leading to muscle wasting (Costamagna et al., 2015; Sharma and Dabur, 2020). Recent advances in secretome studies with post-exercise have revealed the existence of numerous myokines that play beneficial roles in the general health (Pedersen and Akerstrom, 2007; Raschke and Eckel, 2013; So et al., 2014; Eckel, 2019). Multiple cytokines have been identified as potential myokines, and their role and regulatory mechanisms in muscle and body health need to be closely investigated.

### Inflammation in Myogenesis and Muscle Regeneration

Tumor necrosis factor-alpha has been positively and negatively implicated in myoblast differentiation and muscle mass control. TNFα can inhibit myogenic differentiation through nuclear factor kappa B (NF-κB) contributing to muscle wasting (Langen et al., 2004). In addition, TNFα perturbs myoblast cell cycle exit and myogenic differentiation through destabilization of MyoD protein in an NF-κB-dependent manner. Also, it has been reported that TNFα intrinsic to muscle and TNFα secreted by myeloid cells influence myogenic responses and muscle aging (Wang et al., 2018). Transplantation of bone marrow cells from wild-type mice into TNFα-null recipients reduces the number of centrally nucleated myofibers with increased satellite cell numbers; however, sarcopenia is increased under this condition. This effect indicates that myeloid cell-derived TNFα alters muscle cell fusion to aged myofibers, thereby contributing to muscle aging. On the contrary, TNFα promotes myogenic differentiation and muscle regeneration through p38MAPK (Chen et al., 2007). On day 12 post-cardiotoxin (CTX) injury, muscles of TNFR1 and TNFR2 double knockout mice contained immune cells with little signs of regeneration accompanied by reduced levels of Myogenin and p21 relative to normal muscle regeneration in wild-type mice (Chen et al., 2005). In the early stage of muscle injury, TNFα promotes myoblast migration directly through chemotactic activity and indirectly by enhancing matrix metalloproteinase activity at the site of muscle injury (Torrente et al., 2003). These results imply the complex roles of TNFα in the control of muscle regeneration that need to be clarified.

Tumor necrosis factor-like weak inducer of apoptosis (TWEAK) is a cytokine produced by several cell types including muscle and macrophages and is capable of modulating myogenic differentiation (Mittel et al., 2010). C2C12 myoblasts treated with TWEAK exhibit a defect in cell cycle exit concurrent with increased proliferation. TWEAK decreases the muscle-specific gene expression including MyoD and Myogenin, leading to the suppression of myogenic differentiation and myotube formation (Girgenrath et al., 2006). The expression levels of TWEAK and its receptor Fn14 are significantly increased in muscles post-injury, thereby suggesting its role in muscle repair (Mittel et al., 2010). The genetic deletion of TWEAK elevates the expression of embryonic myosin heavy chain and the myofiber cross-sectional area in regenerating tibialis anterior muscles. In addition, TWEAK can induce the expression of several inflammatory molecules and increase the interstitial fibrosis in regenerating muscle. Furthermore, TWEAK ablation suppresses NF-κB activities, without affecting the activation of Akt or p38MAPK in regenerating muscles, while the transgenic expression of TWEAK in myoblasts declines their differentiation capacity compared to wild-type myoblasts, suggesting that TWEAK negatively regulates muscle regeneration. Existing evidence suggests that excessive inflammation impairs muscle regeneration (Al-Sawaf et al., 2014). The activation of NF-κB induced by TWEAK is counterbalanced by Nrf2, a transcription factor that regulates antioxidant enzymes. Nrf2 knockout mice show increased TWEAK expression, enhanced fibrosis, and insufficient regeneration after ischemia/reperfusion muscle injury. In contrast, a low dose of TWEAK activates the non-canonical NF-κB pathway and increases myoblast differentiation at a later stage of myogenic differentiation, without causing atrophy or impairing myogenesis (Enwere et al., 2012). These opposing roles of TWEAK in myoblast differentiation and regeneration processes are related to the concentration-dependent effects of inflammatory cytokines, and further investigations are needed to clarify the mechanisms underlying these opposing activities.

### Inflammatory Cytokines in Muscle Protein Turnover

Proinflammatory cytokines are involved in a complex network that results in the promotion of protein catabolism and suppression of anabolic signaling (Costamagna et al., 2015). In particular, proinflammatory cytokines are well known to impinge on muscle protein turnover. Healthy animals exposed to proinflammatory cytokines, such as TNFα, IL-1, and IL-6, develop muscle wasting through augmenting both ubiquitin expression and proteasome enzymatic activity (Tisdale, 2008). In addition, NF-κB activation and the subsequent MuRF1 induction are critical mechanisms for muscle wasting in response to proinflammatory cytokines, such as TNFα (Cai et al., 2004; Mourkioti and Rosenthal, 2008; Sartori et al., 2021). Furthermore, the action of TNFα on muscle mass can be mediated by p38MAPK/atrogin1/MAFbx induction (Li et al., 2005). TNFα can upregulate Atrogin-1 mRNA in C2C12 myotubes and adult mouse muscle, depending on p38MAPK activation. In addition, TNFα can exert an anti-anabolic effect by downregulating IGF-1 synthesis or by direct interaction with IRS-1, indicating that TNFα might ultimately suppress anabolic signaling through IGF-1 downregulation.

Tumor necrosis factor-like weak inducer of apoptosis treatment significantly decreases PI3K/Akt activation and sequentially downregulates phosphorylation of mTOR and p70S6K, leading to the upregulation of UPS activity in myotubes (Dogra et al., 2007). Soluble TWEAK treatment results in decreased total protein content in C2C12 myotubes. Similarly, mice subjected to chronic administration of soluble TWEAK display reduced fiber diameter and body weight compared to control mice. In the TWEAK-induced myotube degradation, NF-κB signaling is activated and this upregulates the UPS system which is largely responsible for the degradation of myofibrillar proteins through the enzymatic activity of the muscle-specific ubiquitin ligases, Atrogin-1 and MuRF1 (Cai et al., 2004). Further evidence suggests that TWEAK affects muscle mitochondrial contents (Sato et al., 2013; Hindi et al., 2014). TWEAK downregulates both peroxisome proliferator-activated receptor gamma coactivator-1alpha (PGC1α) expression and mitochondrial biogenesis contributing to muscle wasting (Hindi et al., 2014). Levels of PGC1α are significantly increased in the muscle of TWEAK- and Fn14-knockout mice compared to that of wild-type mice in response to denervation. PGC1α hyperexpression attenuates the induction of NF-κB activation and ubiquitin ligases in response to TWEAK leading to the prevention of muscle wasting. Moreover, the age-related loss of muscle oxidative capacity is also attenuated in TWEAK-knockout mice associated with the increased expression of PGC1α and succinate dehydrogenase (Sato et al., 2013).

## PRMTs in Inflammation

**FIGURE 2:**
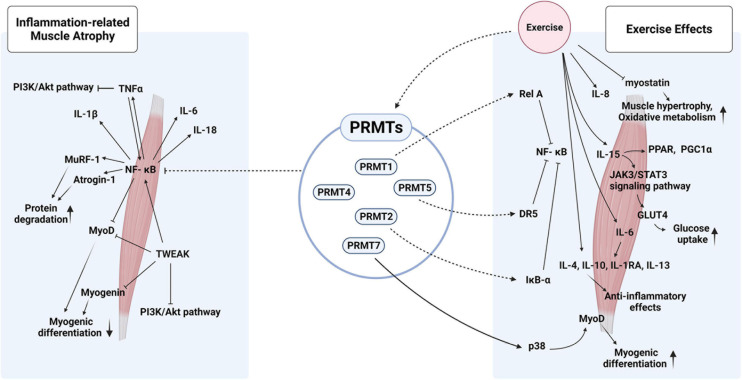
Hypothetical roles of PRMTs in inflammation-related muscle atrophy and exercise-mediated effects on muscle metabolism and hypertrophy. PRMTs could modulate the activities of NK-κB *via* multiple mechanisms to fine-tune the inflammatory responses triggered by pro- and anti-inflammatory cytokines in response to exercise.

The exact roles of PRMTs in muscle inflammation are not well characterized; however, there is a correlation between PRMTs and inflammatory pathways. In particular, PRMTs appear to be modulating NF-κB-mediated processes in the regulation of immune response (Figure 2). NF-κB is a key regulator for the immune and stress responses, often implicated in diseases such as chronic inflammation and cancers (Tak and Firestein, 2001; Liu et al., 2017). PRMT1 appears to be a restrictive factor for NF-κB activation by TNFα. PRMT1 also regulates RelA by arginine methylation, thereby inhibiting the binding of RelA to DNA and inhibiting NF-κB target gene expression in response to TNFα (Reintjes et al., 2016). In addition, the methyltransferase activity of PRMT1 is critical for its coactivator function in context with PRMT4 and CREB-binding proteins (CBP)/p300-binding protein (Hassa et al., 2008). In another context, PRMT1 and NF-κB can act synergistically at the macrophage inflammatory protein 2 and human immunodeficiency virus 1 long terminal repeat promoters. This action is further regulated in concert with CBP/p300-binding protein, PRMT4, and PARP1. In addition, PRMT1 can form a complex with PARP1 and NF-κB *in vivo* and interact with the NF-κB subunit p65 *in vitro*. These reports suggest a complex role of PRMT1 in the control of NF-κB action that needs to be further investigated.

A recent study provides evidence that PRMT4 is a transcriptional coactivator of NF-κB and functions as a promoter-specific regulator of NF-κB recruitment to chromatin (Covic et al., 2005). Interestingly, PRMT4 has been found to be a novel transcriptional coactivator of NF-κB that utilizes a similar set of coactivator proteins with CBP/p300, in p53/nuclear receptor-mediated transcriptional regulation (Covic et al., 2005; Kim et al., 2016). The NF-κB-dependent genes were impaired in TNFα or LPS-stimulated PRMT4 knockout cells. PRMT4 is associated with the formation of NF-κB complex with p300 and p65. It seems that PRMT4 acts as a synergistical coactivator of NF-κB by promoting NF-κB recruitment to cognate sites in a gene-specific manner (Covic et al., 2005). Also, PRMT4 catalyzes CBP/p300 methylation, which is linked to changes in transcription states (Xu et al., 2001). These studies indicate that PRMT1 and PRMT4 can regulate NF-κB-mediated gene expression in a cooperative manner to modulate the inflammatory responses. Further studies are needed to fully understand how PRMT1 and PRMT4 regulate NF-κB activity through their cooperation with other cofactors. PRMT4 seems to interact with and recruit Brg1, an enzymatic ATPase subunit of the SWI/SNF complex, to target genes. In addition, PRMT4 can recruit another interaction partner independent of its enzymatic activities (Kim et al., 2016). These studies suggest that PRMT4 can modulate NF-κB-dependent gene expression *via* multiple mechanisms that need to be further clarified.

In addition to PRMT1 and PRMT4, PRMT2 has also been shown to play a role in the inflammatory pathway by opposing NF-κB-dependent transcription upon TNFα signaling by sequestering the NF-κB inhibitor IκB-α in the nucleus where NF-κB functions (Ganesh et al., 2006; Dalloneau et al., 2011). PRMT2 has been considered as a new member of the NF-κB pathway controlling LPS-induced inflammatory and lung responses by regulating the nuclear accumulation of NF-κB in fibroblasts (Dalloneau et al., 2011).

PRMT5 appears to be associated with the immune regulation mediated by TNF-related apoptosis-inducing ligand (TRAIL) receptor 1 by controlling NF-κB activities (Wang S.C.M. et al., 2012). Additionally, the regulation of NF-κB activities by PRMT5 is implicated in TRAIL-induced apoptosis (Tanaka et al., 2009). Binding of PRMT5 to the TRAIL receptor stimulates IKK activation and IκB degradation, indicating that PRMT5 is involved in inflammatory responses. Furthermore, PRMT5 can directly regulate NF-κB activity by inducing methylation of the p65 subunit, thereby regulating NF-κB-dependent gene expressions, such as IL-1α and TNF receptor-associated factor 1. Methylation of p65 at Arg30 and Arg35 by PRMT5 elevates p65 levels and the transcription of a subset of TNFα-induced proinflammatory genes (Wei et al., 2013; Harris et al., 2014). Finally, PRMT7-mediated arginine methylation regulates the genes involved in mammalian immune and inflammatory responses, and the deletion of PRMT7 in B cells impairs B-cell differentiation and induces an immune deregulation reminiscent of sterile inflammation (Ying et al., 2015). Based on the current knowledge on PRMTs in the modulation of NF-κB activity and inflammation, PRMTs might play critical roles in the modulation of muscle inflammation responses as muscle intrinsic factor. Further studies are required to address these specific aspects of the role of PRMT.

## Exercise, PRMTs, and Inflammation

Exercise initiates a cascade of inflammatory events, which ultimately lead to long-term effects on human health (Allen et al., 2015). The benefits of physical exercise include protection against chronic diseases such as cardiovascular diseases, cancer, chronic respiratory diseases, and type II diabetes mellitus (Gleeson et al., 2011; Leal et al., 2018). Multiple studies have shown that physical exercise reduces blood pressure, increases cardiac function, and improves glucose homeostasis and insulin sensitivity (Goodyear and Kahn, 1998; Barauna et al., 2005; Hegde and Solomon, 2015; Madonna et al., 2020). Physical activity reduces the risk of age-associated diseases and improves the inflammatory profile (Beavers et al., 2010; Sparling et al., 2015). In addition, physical exercise protects the muscles against disease-induced muscle wasting, and the expression and activity of PRMTs are modulated in the muscles in response to exercise (Figure 2). The PRMT5-specific activity increases during the post-exercise recovery period, thereby providing support in muscle plasticity post-exercise (Blanc and Richard, 2017). The expression, localization, and activity of PRMT1, PRMT4, and PRMT5 are altered in muscles upon denervation, thereby suggesting their role in disuse-induced muscle remodeling (Stouth et al., 2018). Investigations have been carried out on the alteration in the levels and activities of PRMTs in acute and chronic training/exercise-induced skeletal muscle remodeling in humans (Vanlieshout et al., 2018, 2019). Interestingly, the expression and activity of PRMTs are distinctively regulated in response to acute or chronic exercises. In acute training, PRMT1, CARM1, and PRMT5 are significantly increased in the muscle while PRMT7 and PRMT9 remain unchanged. In the chronic exercise models, the participants are subjected to sprint interval training (SIT) and quadriceps muscle biopsies at resting muscle (PRE), mid-training (MID), and post-training (POST) are taken. The results reveal that there is no great alteration in the level of PRMT1, PRMT5, and PRMT7 proteins while CARM1/PRMT4 proteins are elevated by about 20% at the MID point relative to that of PRE. In addition, PRMT-specific methyltransferase activities related to PRMT1, CARM1/PRMT4, and PRMT5 are also differently altered in response to chronic exercise. It is evident that chronic exercise can modulate PRMTs at their expression as well as enzyme activity. Similar to PRMTs, PGC1α is differentially regulated by acute and chronic exercise. PGC1α is elevated in skeletal muscle after a single bout of exercise (Little et al., 2011), while it does not change post chronic training (Little et al., 2010; Edgett et al., 2016). Since PGC1α physically interacts with PRMT1 or PRMT4/CARM1 in the muscle (Vanlieshout et al., 2018), these proteins could be involved in the enhanced mitochondrial biogenesis and activities mediated by exercise. Taken together, these studies suggest the potential roles of PRMTs in exercise-induced muscle remodeling and other health benefits associated with chronic exercise. Further studies are required to elucidate the exact role and mechanisms of PRMTs in exercise-mediated muscle plasticity.

Proinflammatory cytokines have been implicated in exercise-mediated, long-term adaptive responses. Inflammatory responses have been shown to contribute to the repair/remodeling processes post-injury or other stimuli such as exercise (Oishi and Manabe, 2018). In response to exercise, the muscle expresses several cytokines such as TNFα, IL-6, IL-1β, IL-4, IL-1RA, IL-8, IL-13, IL-10, IL-15, and leukemia inhibitory factor (Pedersen, 2013), and these muscle contraction-mediated myokines can play a role in pro- and anti-inflammatory effects of exercise, contributing to health benefits (Brandt and Pedersen, 2010; Severinsen and Pedersen, 2020). After exercise training, the proinflammatory cytokines are initially released followed by the release of anti-inflammatory cytokines that attenuate the proinflammatory responses (Moldoveanu et al., 2001). Post muscle contraction, the muscles secrete myokines, termed as “exercise factor” (Pedersen, 2011), which trigger metabolic and physiological responses in other organs (Pedersen et al., 2003; Leal et al., 2018).

Among myokines, IL-6 is the first identified and the most studied myokine that is produced by contracting muscles. IL-6 can exert both pro- and anti-inflammatory effects depending on the context. IL-6 is generally classified as a proinflammatory cytokine and chronically elevated levels of IL-6 exert proinflammatory effects, likely contributing to muscle wasting related to aging or other muscle diseases. The basis of the chronic elevation of IL-6 and its regulatory mechanisms are currently unclear. Upon exercise, the acute increase of IL-6 levels produced by contracting muscles plays a beneficial role in muscle function and systemic anti-inflammation. The acute increase in IL-6 levels upon exercise stimulates the subsequent production of anti-inflammatory cytokines, IL-1 receptor antagonist (IL-1RA) and IL-10, thereby generating an anti-inflammatory systemic environment and the consequent exercise effect (Steensberg et al., 2003; Tanaka et al., 2014). Exercise and intravenous infusion of IL-6 protect the acute inflammation by elevating plasma IL-10, IL-1RA, and cortisol and inhibiting endotoxin-stimulated TNFα levels (Starkie et al., 2003; Steensberg et al., 2003). IL-1RA inhibits IL-1β signaling pathway (Dinarello, 1994), and IL-10 downregulates TNFα synthesis (Opp et al., 1995).

In response to an ultra-endurance exercise bout in experienced athletes, the secretion of IL-8 from muscle is altered (Marklund et al., 2013). Interestingly, overproduction of IL-8 is found in myotubes from patients with type II diabetes and is associated with affecting muscle capillarization, thereby reducing muscle exposure to glucose. Thus, IL-8 overproduction seems to impinge on muscle wasting and the primary disease itself (Levy et al., 2015). In addition, IL-15 expression is induced in muscle cells by physical exercise (Ye, 2015; Gonzalez-Gil and Elizondo-Montemayor, 2020). IL-15 increases glucose uptake in muscle and improves peroxisome proliferator-activated receptor and PGC1α activity, thereby favoring mitochondrial biogenesis and fatty acid oxidation (Quinn et al., 2014; Sun and Liu, 2015; Krolopp et al., 2016). IL-15 concentrations in muscle biopsy are markedly increased despite no apparent increase in the circulation following exercise (Rinnov et al., 2014). Furthermore, IL-15 promotes muscle hypertrophy, facilitating the utilization of free fatty acids, thereby preventing their reincorporation to visceral adipose tissue.

The relationship between cytokines and PRMTs is largely unknown. Two recent studies have reported the role of PRMT1 and PRMT5 in IL-6 and IL-2 in non-muscle system, respectively. In macrophages, PRMT1 plays a critical role in IL-6 production (Zhao et al., 2019), while PRMT5 is important for IL-2 expression in T-cell activation (Richard et al., 2005). The fact that the expression and activity of PRMTs are regulated in exercised muscles suggest that PRMTs might be involved in the regulation of myokine production in response to muscle activities. In addition, the regulation of TNFα/NF-κB signaling by PRMTs might be critical to fine-tuning the pro- and anti-inflammatory responses in muscles during regeneration and aging environment. Future investigations are needed to enlighten the precise roles of PRMTs in muscle inflammatory responses and myokine production. It is essential to understand the molecular regulatory mechanisms controlling inflammation to develop novel targets for the therapeutic intervention of muscle wasting related to aging or chronic inflammation.

## Conclusion

Despite the current limitation in the number of studies investigating the function of PRMTs in the muscle, accumulating evidence strongly suggests that this family of enzymes is emerging as key players in the regulation of muscle homeostasis and plasticity. PRMTs have been implicated in muscle development and regeneration and also in the control of glucose/oxidative muscle metabolism. In consideration of the fact that diverse muscle diseases are tightly linked with perturbed muscle regeneration capacity and muscle metabolism, PRMTs can be targeted to improve muscle regeneration capacity and metabolic health related to muscle diseases. Although more detailed analyses are required, multiple studies have proposed the potential role of PRMTs in muscle remodeling in response to acute and long-term exercise, suggestive of their role in mediating the effects of exercise by the regulation of gene expression and signaling pathways. Thus, enhancing the activity of PRMTs appears to be a tentative strategy to maximize the effects of exercise on muscle and metabolic health. In consideration of the suppressive role of multiple PRMTs in NF-κB activity in non-muscle cell types, it can be speculated that boosting the activity of PRMTs might suppress NF-κB activity and MuRF1 induction triggered by proinflammatory cytokines and the resulting muscle wasting. Thus, we believe that PRMTs have the potential as therapeutic targets to prevent muscle wasting related to aging or other chronic diseases.

## Author Contributions

H-KS, SK, J-SK, and S-JL conceptualized and drafted the manuscript. All authors revised the work and final approval of the manuscript.

## Conflict of Interest

H-KS and S-JL are employed by AniMusCure Inc. The remaining authors declare that the research was conducted in the absence of any commercial or financial relationships that could be construed as a potential conflict of interest.

## Publisher’s Note

All claims expressed in this article are solely those of the authors and do not necessarily represent those of their affiliated organizations, or those of the publisher, the editors and the reviewers. Any product that may be evaluated in this article, or claim that may be made by its manufacturer, is not guaranteed or endorsed by the publisher.
